# 1-[(*E*)-(2-Methyl-3-nitro­phen­yl)imino­meth­yl]-2-naphthol

**DOI:** 10.1107/S1600536810043461

**Published:** 2010-11-06

**Authors:** Yelda Bingöl Alpaslan, Ayşen Alaman Ağar, Şamil Işık

**Affiliations:** a Department of Physics, Faculty of Arts and Sciences, Ondokuz Mayıs University, TR-55139 Kurupelit-Samsun, Turkey; b Department of Chemistry, Faculty of Arts and Sciences, Ondokuz Mayıs University, 55139 Samsun, Turkey

## Abstract

The title Schiff base compound, C_18_H_14_N_2_O_3_, has an inter­mediate state between NH and OH tautomers. The mol­ecular structure is stabilized by an O—H⋯N hydrogen bond. The dihedral angle between the naphthalene ring system and the benzene ring is 37.44 (5)°.

## Related literature

For the biological properties of Schiff bases, see: Lozier *et al.* (1975 ▶). For the coordination chemistry of Schiff bases, see: Kargar *et al.* (2009 ▶); Yeap *et al.* (2009 ▶). For Schiff base tautomerism, see: Hökelek *et al.* (2000 ▶); Karabıyık *et al.* (2007 ▶); Odabaşoğlu *et al.* (2005 ▶); Kılıç *et al.* (2009 ▶). For bond-length data, see: Allen *et al.* (1987 ▶).
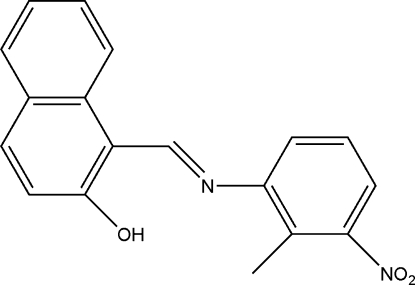


## Experimental

### 

#### Crystal data

C_18_H_14_N_2_O_3_
*M* *_r_* = 306.31
Monoclinic, 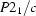

*a* = 12.5520 (9) Å
*b* = 7.4731 (4) Å
*c* = 15.8610 (13) Å
β = 90.806 (6)°
*V* = 1487.65 (18) Å^3^
*Z* = 4
Mo *K*α radiation
μ = 0.10 mm^−1^
*T* = 296 K
0.7 × 0.47 × 0.12 mm

#### Data collection

Stoe IPDS II diffractometer
Absorption correction: integration (*X-RED32*; Stoe & Cie, 2002 ▶) *T*_min_ = 0.989, *T*_max_ = 0.997
9684 measured reflections
3088 independent reflections
1969 reflections with *I* > 2σ(*I*)
*R*_int_ = 0.061

#### Refinement

*R*[*F* ^2^ > 2σ(*F* ^2^)] = 0.051
*wR*(*F* ^2^) = 0.153
*S* = 0.94
3088 reflections
213 parameters
H atoms treated by a mixture of independent and constrained refinement
Δρ_max_ = 0.24 e Å^−3^
Δρ_min_ = −0.19 e Å^−3^

### 

Data collection: *X-AREA* (Stoe & Cie, 2002 ▶); cell refinement: *X-AREA*; data reduction: *X-RED32* (Stoe & Cie, 2002 ▶); program(s) used to solve structure: *SHELXS97* (Sheldrick, 2008 ▶); program(s) used to refine structure: *SHELXL97* (Sheldrick, 2008 ▶); molecular graphics: *ORTEP-3 for Windows* (Farrugia, 1997 ▶); software used to prepare material for publication: *WinGX* (Farrugia, 1999 ▶).

## Supplementary Material

Crystal structure: contains datablocks I, global. DOI: 10.1107/S1600536810043461/bt5391sup1.cif

Structure factors: contains datablocks I. DOI: 10.1107/S1600536810043461/bt5391Isup2.hkl

Additional supplementary materials:  crystallographic information; 3D view; checkCIF report

## Figures and Tables

**Table 1:** Hydrogen-bond geometry (Å, °)

*D*—H⋯*A*	*D*—H	H⋯*A*	*D*⋯*A*	*D*—H⋯*A*
O1—H1⋯N1	1.10 (3)	1.48 (3)	2.5310 (19)	159 (3)

**Fig. 1.**
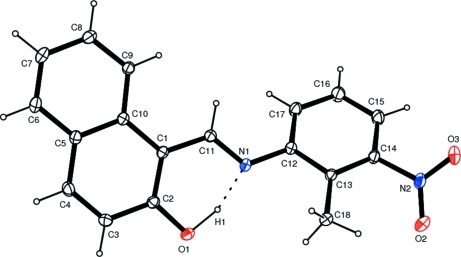
The molecular structure of the title compound, showing the atom-numbering scheme. Displacement ellipsoids are drawn at the 30% probability level.

## supplementary crystallographic information

### Comment

Schiff bases often exhibit various biological activities and in many cases were shown to have antibacterial, anticancer, anti-inflammatory and antitoxic properties (Lozier *et al.*, 1975). Schiff bases have also been used as versatile ligands in coordination chemistry (Kargar *et al.*, 2009; Yeap *et al.*, 2009). There are two types of intramolecular hydrogen bonds in Schiff bases, namely N—H···O in keto (NH) (Hökelek *et al.*, 2000) and N···H—O in enol (OH) (Odabaşoǧlu *et al.*, 2005) tautomeric forms. Our investigations shows that in the title compound is described as an intermediate state between NH and OH tautomers. An *ORTEP-3* (Farrugia, 1997) plot of the molecule of (I) is shown in Fig.1. The C2—O1 [1.331 (2) Å] and C11—N1 [1.299 (2) Å] bond lengths are intermediate between the single and double C—O (1.362 and 1.222 Å, respectively) and C—N bond lengths (1.339 and 1.279 Å, respectively) (Allen *et al.*, 1987). The molecular structure is stabilized by an intramolecular O—H···N hydrogen bond. It is a well known fact that H atoms participating in intramolecular hydrogen bonds in Schiff bases are rather mobile. The molecule can be regarded as having an intermediate state between its canonical OH and NH forms, and therefore the O1—H1 bond [1.10 (3) Å] remains somewhat longer than its expected value (Karabıyık *et al.*, 2007). Similar results were observed for 2-[(4-Methoxyphenyl)iminomethyl]-4- nitrophenol (Kılıç *et al.*, 2009). The molecule of the title compound is not planar, with a dihedral angle of 37.44 (5)° between naphthalene and benzene rings.

### Experimental

The title compound was prepared by refluxing a mixture of a solution containing 2-hydroxy-1-naphthaldehyde (172 mg, 0,0986 mmol) in ethanol (30 ml) and a solution containing 2-methyl-3-nitroaniline (15 mg, 0,0986 mmol) in ethanol (30 ml. The reaction mixture was stirred for 1 h under reflux. Single crystals of the title compound for *x*-ray analysis were obtained by slow evaporation of an ethanol solution (Yield 68%; m.p. 428–432 K).

### Refinement

H atoms bonded to C were placed in calculated positions and constrained to ride on their parent atoms, with C–H = 0.93–0.96 Å and *U*_iso_(H) = 1.2 *U*_eq_(C) or *U*_iso_(H) = 1.5 *U*_eq_(C_methyl_). The H atom bonded to O was freely refined.

### Crystal data

**Table:**

C_18_H_14_N_2_O_3_	*F*(000) = 640
*M**_r_* = 306.31	*D*_x_ = 1.368 Mg m^−^^3^
Monoclinic, *P*2_1_/*c*	Mo *K*α radiation, λ = 0.71073 Å
Hall symbol: -P 2ybc	Cell parameters from 8237 reflections
*a* = 12.5520 (9) Å	θ = 1.3–27.3°
*b* = 7.4731 (4) Å	µ = 0.10 mm^−^^1^
*c* = 15.8610 (13) Å	*T* = 296 K
β = 90.806 (6)°	PRISM., yellow
*V* = 1487.65 (18) Å^3^	0.7 × 0.47 × 0.12 mm
*Z* = 4	

### Data collection

**Table:**

Stoe IPDS II diffractometer	3088 independent reflections
Radiation source: fine-focus sealed tube	1969 reflections with *I* > 2σ(*I*)
graphite	*R*_int_ = 0.061
Detector resolution: 6.67 pixels mm^-1^	θ_max_ = 26.5°, θ_min_ = 1.6°
ω scans	*h* = −15→15
Absorption correction: integration (*X-RED32*; Stoe & Cie, 2002)	*k* = −9→9
*T*_min_ = 0.989, *T*_max_ = 0.997	*l* = −19→17
9684 measured reflections	

### Refinement

**Table:**

Refinement on *F*^2^	Secondary atom site location: difference Fourier map
Least-squares matrix: full	Hydrogen site location: inferred from neighbouring sites
*R*[*F*^2^ > 2σ(*F*^2^)] = 0.051	H atoms treated by a mixture of independent and constrained refinement
*wR*(*F*^2^) = 0.153	*w* = 1/[σ^2^(*F*_o_^2^) + (0.095*P*)^2^] where *P* = (*F*_o_^2^ + 2*F*_c_^2^)/3
*S* = 0.94	(Δ/σ)_max_ < 0.001
3088 reflections	Δρ_max_ = 0.24 e Å^−^^3^
213 parameters	Δρ_min_ = −0.19 e Å^−^^3^
0 restraints	Extinction correction: *SHELXL97* (Sheldrick, 2008), Fc^*^=kFc[1+0.001xFc^2^λ^3^/sin(2θ)]^-1/4^
Primary atom site location: structure-invariant direct methods	Extinction coefficient: 0.009 (2)

### Special details

**Table:**

Geometry. All e.s.d.'s (except the e.s.d. in the dihedral angle between two l.s. planes) are estimated using the full covariance matrix. The cell e.s.d.'s are taken into account individually in the estimation of e.s.d.'s in distances, angles and torsion angles; correlations between e.s.d.'s in cell parameters are only used when they are defined by crystal symmetry. An approximate (isotropic) treatment of cell e.s.d.'s is used for estimating e.s.d.'s involving l.s. planes.
Refinement. Refinement of *F*^2^ against ALL reflections. The weighted *R*-factor *wR* and goodness of fit *S* are based on *F*^2^, conventional *R*-factors *R* are based on *F*, with *F* set to zero for negative *F*^2^. The threshold expression of *F*^2^ > σ(*F*^2^) is used only for calculating *R*-factors(gt) *etc*. and is not relevant to the choice of reflections for refinement. *R*-factors based on *F*^2^ are statistically about twice as large as those based on *F*, and *R*- factors based on ALL data will be even larger.

### Fractional atomic coordinates and isotropic or equivalent isotropic displacement parameters (Å^2^)

**Table:**

	*x*	*y*	*z*	*U*_iso_*/*U*_eq_	
C1	0.41741 (13)	0.0570 (2)	0.21696 (11)	0.0439 (4)	
C2	0.35899 (13)	0.0014 (2)	0.28723 (11)	0.0489 (4)	
C3	0.24646 (15)	−0.0112 (3)	0.28256 (13)	0.0605 (5)	
H3	0.2085	−0.0498	0.3291	0.073*	
C4	0.19459 (14)	0.0329 (3)	0.21045 (13)	0.0627 (5)	
H4	0.1206	0.0258	0.2086	0.075*	
C5	0.24883 (13)	0.0898 (3)	0.13724 (12)	0.0510 (4)	
C6	0.19243 (15)	0.1360 (3)	0.06252 (14)	0.0618 (5)	
H6	0.1184	0.1309	0.0616	0.074*	
C7	0.24441 (16)	0.1873 (3)	−0.00749 (14)	0.0648 (5)	
H7	0.2062	0.2181	−0.0560	0.078*	
C8	0.35511 (16)	0.1941 (3)	−0.00694 (13)	0.0606 (5)	
H8	0.3907	0.2270	−0.0556	0.073*	
C9	0.41227 (14)	0.1526 (3)	0.06482 (11)	0.0515 (5)	
H9	0.4862	0.1594	0.0642	0.062*	
C10	0.36138 (12)	0.1001 (2)	0.13924 (11)	0.0441 (4)	
C11	0.53047 (13)	0.0720 (2)	0.22409 (12)	0.0471 (4)	
H11	0.5686	0.1078	0.1772	0.057*	
C12	0.69421 (13)	0.0479 (2)	0.29892 (11)	0.0487 (4)	
C13	0.74005 (13)	0.1057 (2)	0.37579 (11)	0.0461 (4)	
C14	0.85070 (14)	0.1110 (3)	0.37726 (12)	0.0557 (5)	
C15	0.91392 (15)	0.0627 (4)	0.31127 (15)	0.0757 (7)	
H15	0.9877	0.0702	0.3159	0.091*	
C16	0.86568 (16)	0.0027 (4)	0.23779 (15)	0.0813 (7)	
H16	0.9068	−0.0323	0.1924	0.098*	
C17	0.75641 (15)	−0.0048 (3)	0.23229 (13)	0.0655 (6)	
H17	0.7239	−0.0461	0.1830	0.079*	
C18	0.67164 (14)	0.1657 (3)	0.44710 (12)	0.0562 (5)	
H18A	0.7161	0.2005	0.4940	0.084*	
H18B	0.6260	0.0692	0.4637	0.084*	
H18C	0.6290	0.2657	0.4292	0.084*	
N1	0.58208 (11)	0.0375 (2)	0.29377 (9)	0.0481 (4)	
N2	0.90826 (13)	0.1746 (3)	0.45348 (12)	0.0695 (5)	
O1	0.40602 (11)	−0.0411 (2)	0.36024 (8)	0.0609 (4)	
O2	0.88124 (15)	0.1224 (3)	0.52187 (11)	0.1036 (7)	
O3	0.98286 (12)	0.2763 (3)	0.44235 (12)	0.0997 (6)	
H1	0.489 (3)	−0.023 (4)	0.343 (2)	0.128 (10)*	

### Atomic displacement parameters (Å^2^)

**Table:**

	*U*^11^	*U*^22^	*U*^33^	*U*^12^	*U*^13^	*U*^23^
C1	0.0467 (8)	0.0410 (9)	0.0437 (9)	0.0020 (7)	−0.0038 (7)	−0.0022 (8)
C2	0.0532 (9)	0.0499 (10)	0.0435 (10)	0.0026 (7)	0.0003 (7)	−0.0048 (8)
C3	0.0548 (10)	0.0759 (14)	0.0510 (11)	−0.0020 (9)	0.0092 (8)	−0.0036 (10)
C4	0.0419 (9)	0.0810 (14)	0.0652 (13)	0.0029 (9)	0.0014 (9)	−0.0089 (11)
C5	0.0472 (9)	0.0541 (11)	0.0515 (11)	0.0041 (7)	−0.0059 (8)	−0.0083 (9)
C6	0.0506 (10)	0.0709 (13)	0.0634 (13)	0.0104 (9)	−0.0158 (9)	−0.0083 (11)
C7	0.0716 (12)	0.0672 (13)	0.0552 (12)	0.0102 (10)	−0.0218 (10)	−0.0007 (10)
C8	0.0738 (12)	0.0603 (12)	0.0474 (11)	−0.0026 (9)	−0.0073 (9)	0.0052 (9)
C9	0.0531 (9)	0.0517 (10)	0.0496 (11)	−0.0050 (8)	−0.0061 (8)	0.0033 (9)
C10	0.0478 (9)	0.0391 (9)	0.0453 (9)	0.0011 (7)	−0.0044 (7)	−0.0029 (7)
C11	0.0493 (9)	0.0461 (10)	0.0459 (10)	−0.0011 (7)	−0.0026 (7)	0.0003 (8)
C12	0.0470 (9)	0.0509 (10)	0.0480 (10)	0.0015 (7)	−0.0048 (7)	−0.0003 (8)
C13	0.0473 (8)	0.0476 (10)	0.0434 (9)	−0.0002 (7)	−0.0050 (7)	0.0031 (8)
C14	0.0487 (9)	0.0655 (12)	0.0526 (11)	0.0008 (8)	−0.0113 (8)	−0.0018 (9)
C15	0.0443 (10)	0.1099 (19)	0.0727 (15)	0.0034 (10)	−0.0026 (9)	−0.0122 (14)
C16	0.0546 (11)	0.122 (2)	0.0674 (14)	0.0087 (12)	0.0058 (10)	−0.0223 (15)
C17	0.0570 (10)	0.0860 (15)	0.0533 (12)	0.0069 (10)	−0.0049 (9)	−0.0168 (11)
C18	0.0567 (10)	0.0636 (12)	0.0481 (10)	−0.0007 (8)	−0.0018 (8)	0.0019 (9)
N1	0.0471 (8)	0.0497 (8)	0.0473 (8)	0.0010 (6)	−0.0083 (6)	−0.0003 (7)
N2	0.0517 (9)	0.0915 (14)	0.0646 (12)	0.0002 (9)	−0.0173 (8)	−0.0044 (11)
O1	0.0635 (8)	0.0769 (10)	0.0422 (7)	0.0031 (7)	−0.0009 (6)	0.0051 (7)
O2	0.1009 (13)	0.154 (2)	0.0552 (10)	−0.0192 (12)	−0.0220 (9)	0.0058 (11)
O3	0.0650 (9)	0.1341 (17)	0.0991 (13)	−0.0299 (10)	−0.0239 (9)	−0.0091 (12)

### Geometric parameters (Å, °)

**Table:**

C1—C2	1.406 (2)	C11—H11	0.9300
C1—C11	1.426 (2)	C12—C17	1.380 (3)
C1—C10	1.447 (2)	C12—C13	1.409 (2)
C2—O1	1.331 (2)	C12—N1	1.411 (2)
C2—C3	1.417 (3)	C13—C14	1.389 (2)
C3—C4	1.349 (3)	C13—C18	1.498 (3)
C3—H3	0.9300	C14—C15	1.371 (3)
C4—C5	1.420 (3)	C14—N2	1.478 (3)
C4—H4	0.9300	C15—C16	1.381 (3)
C5—C6	1.415 (3)	C15—H15	0.9300
C5—C10	1.415 (2)	C16—C17	1.374 (3)
C6—C7	1.351 (3)	C16—H16	0.9300
C6—H6	0.9300	C17—H17	0.9300
C7—C8	1.390 (3)	C18—H18A	0.9600
C7—H7	0.9300	C18—H18B	0.9600
C8—C9	1.372 (3)	C18—H18C	0.9600
C8—H8	0.9300	N2—O2	1.206 (2)
C9—C10	1.406 (2)	N2—O3	1.221 (2)
C9—H9	0.9300	O1—H1	1.10 (3)
C11—N1	1.299 (2)		
			
C2—C1—C11	119.33 (16)	C1—C11—H11	118.8
C2—C1—C10	119.26 (15)	C17—C12—C13	121.45 (16)
C11—C1—C10	121.41 (15)	C17—C12—N1	120.96 (16)
O1—C2—C1	122.05 (15)	C13—C12—N1	117.52 (15)
O1—C2—C3	117.35 (16)	C14—C13—C12	114.78 (16)
C1—C2—C3	120.60 (16)	C14—C13—C18	124.22 (16)
C4—C3—C2	119.81 (18)	C12—C13—C18	120.91 (15)
C4—C3—H3	120.1	C15—C14—C13	124.69 (17)
C2—C3—H3	120.1	C15—C14—N2	115.32 (16)
C3—C4—C5	122.38 (17)	C13—C14—N2	119.98 (17)
C3—C4—H4	118.8	C14—C15—C16	118.58 (18)
C5—C4—H4	118.8	C14—C15—H15	120.7
C6—C5—C10	119.57 (18)	C16—C15—H15	120.7
C6—C5—C4	121.22 (16)	C17—C16—C15	119.5 (2)
C10—C5—C4	119.21 (16)	C17—C16—H16	120.3
C7—C6—C5	121.06 (17)	C15—C16—H16	120.3
C7—C6—H6	119.5	C16—C17—C12	120.96 (19)
C5—C6—H6	119.5	C16—C17—H17	119.5
C6—C7—C8	119.95 (17)	C12—C17—H17	119.5
C6—C7—H7	120.0	C13—C18—H18A	109.5
C8—C7—H7	120.0	C13—C18—H18B	109.5
C9—C8—C7	120.52 (19)	H18A—C18—H18B	109.5
C9—C8—H8	119.7	C13—C18—H18C	109.5
C7—C8—H8	119.7	H18A—C18—H18C	109.5
C8—C9—C10	121.39 (17)	H18B—C18—H18C	109.5
C8—C9—H9	119.3	C11—N1—C12	121.57 (15)
C10—C9—H9	119.3	C11—N1—H1	97.0 (12)
C9—C10—C5	117.49 (16)	C12—N1—H1	141.0 (12)
C9—C10—C1	123.80 (15)	O2—N2—O3	123.89 (19)
C5—C10—C1	118.71 (16)	O2—N2—C14	119.41 (19)
N1—C11—C1	122.38 (17)	O3—N2—C14	116.68 (19)
N1—C11—H11	118.8	C2—O1—H1	99.5 (17)
			
C11—C1—C2—O1	−1.2 (3)	C2—C1—C11—N1	−0.6 (3)
C10—C1—C2—O1	179.59 (16)	C10—C1—C11—N1	178.56 (17)
C11—C1—C2—C3	178.70 (17)	C17—C12—C13—C14	2.2 (3)
C10—C1—C2—C3	−0.5 (3)	N1—C12—C13—C14	179.22 (16)
O1—C2—C3—C4	178.99 (19)	C17—C12—C13—C18	178.92 (19)
C1—C2—C3—C4	−0.9 (3)	N1—C12—C13—C18	−4.1 (3)
C2—C3—C4—C5	1.0 (3)	C12—C13—C14—C15	−0.9 (3)
C3—C4—C5—C6	−179.8 (2)	C18—C13—C14—C15	−177.5 (2)
C3—C4—C5—C10	0.4 (3)	C12—C13—C14—N2	178.16 (18)
C10—C5—C6—C7	1.0 (3)	C18—C13—C14—N2	1.6 (3)
C4—C5—C6—C7	−178.9 (2)	C13—C14—C15—C16	−0.6 (4)
C5—C6—C7—C8	0.5 (3)	N2—C14—C15—C16	−179.7 (2)
C6—C7—C8—C9	−1.4 (3)	C14—C15—C16—C17	0.8 (4)
C7—C8—C9—C10	0.8 (3)	C15—C16—C17—C12	0.5 (4)
C8—C9—C10—C5	0.6 (3)	C13—C12—C17—C16	−2.1 (3)
C8—C9—C10—C1	−179.21 (17)	N1—C12—C17—C16	−179.0 (2)
C6—C5—C10—C9	−1.5 (3)	C1—C11—N1—C12	178.07 (15)
C4—C5—C10—C9	178.39 (18)	C17—C12—N1—C11	−37.0 (3)
C6—C5—C10—C1	178.35 (17)	C13—C12—N1—C11	145.97 (18)
C4—C5—C10—C1	−1.8 (3)	C15—C14—N2—O2	−135.8 (2)
C2—C1—C10—C9	−178.35 (16)	C13—C14—N2—O2	45.0 (3)
C11—C1—C10—C9	2.5 (3)	C15—C14—N2—O3	42.8 (3)
C2—C1—C10—C5	1.8 (2)	C13—C14—N2—O3	−136.3 (2)
C11—C1—C10—C5	−177.34 (15)		

### Hydrogen-bond geometry (Å, °)

**Table:**

*D*—H···*A*	*D*—H	H···*A*	*D*···*A*	*D*—H···*A*
O1—H1···N1	1.10 (3)	1.48 (3)	2.5310 (19)	159 (3)
